# Twist 1 regulates the expression of PPARγ during hormone-induced 3T3-L1 preadipocyte differentiation: a possible role in obesity and associated diseases

**DOI:** 10.1186/1476-511X-13-132

**Published:** 2014-08-16

**Authors:** Wanshan Ma, Sumei Lu, Tao Sun, Xiangdong Wang, Yongmei Ma, Xiaoli Zhang, Ruxing Zhao, Yunshan Wang

**Affiliations:** Department of Laboratory Medicine, Shandong Provincial Qianfoshan Hospital, Shandong University, Jingshi Road 16766, Jinan, Shandong 250014 P. R. China; Medical Research & Laboratory Diagnostic Center, Jinan Center Hospital Affiliated to Shandong University, Jinan, Shandong 250013 P.R. China; The Institute of Cell Biology, Shandong University School of Medicine, Jinan, Shandong 250012 P.R. China; Department of Endocrinology, Department of Nephrology, Qilu Hospital of Shandong University, Jinan, Shandong 250012 P.R. China

**Keywords:** Twist 1, Obesity, PPARγ, Adipocyte differentiation, Adipokines

## Abstract

**Background:**

Twist 1 is highly expressed in adipose tissue and has been associated with obesity and related disorders. However, the molecular function of Twist 1 in adipose tissue is unclear. Twist 1 has been implicated in cell lineage determination and differentiation. Therefore, we investigated both the role of Twist 1 in adipocyte precursor mobilization and the relationship of Twist 1 with other molecular determinants of adipocyte differentiation.

**Methods:**

We examined Twist 1 mRNA and protein expression in subcutaneous adipose tissues from diet-induced obese C57/BL6 mice and Wistar rats and in obese patients undergoing liposuction or adipose transplant surgeries. Twist 1 expression was measured on days 0, 2, 4, 8, and 12 of 3T3-L1 differentiation in vitro. The role of Twist 1 in adipogenesis was explored using retroviral interference of Twist 1 expression. Adipokine secretion was evaluated using a RayBio® Biotin Label-based Adipokine Array.

**Results:**

Twist 1 mRNA and protein levels were reduced in diet-induced obese mice and rats and in obese humans. Twist 1 was upregulated during 3T3-L1 preadipocyte differentiation in vitro, beginning from the fourth day of differentiation induction. Retroviral interference of Twist 1 expression did not significantly impair lipid formation; however, retroviral interference induced PPARγ mRNA and protein expression on day 4 of differentiation induction. Adipokine array analyses revealed increased secretion of CXCR4 (19.55-fold), VEGFR1 (92.13-fold), L-21 R (63.55-fold), and IL-12 R beta 1 (59.66-fold) and decreased secretion of VEGFR3 (0.01-fold), TSLP R (0.071-fold), MIP-1 gamma (0.069-fold), TNF RI/TNFRSF1A (0.09-fold), and MFG-E8 (0.06-fold).

**Conclusions:**

Twist 1 is a regulator of adipocyte gene expression although it is not likely to regulate differentiation. We identified PPARγ as a potential target of Twist 1 and found variation in the secretion of multiple adipokines, which might indicate a prospective mechanism linking Twist 1 expression with obesity or associated diseases.

## Background

Obesity has become an epidemic in the human population, and China has the highest number of obese patients in the world [1]. Because obesity involves an increase in the number of adipocytes, any of the factors involved in adipocyte differentiation might be of great importance for the development of obesity. To date, numerous factors and proteins have been implicated in the generation of new fat cells, including peroxisome proliferator-activated receptor gamma (PPARγ) [2, 3], CCAAT/enhancer binding protein (C/EBP, which includes C/EBP α, C/EBP β, and C/EBP δ) [4, 5], adipocyte lipid binding protein (ALBP), and adipocyte determination and differentiation factor 1 (ADD1) [6, 7]. However, the relevance of those factors in the development of obesity remains unclear.

Evidence suggests that PPARγ is a key player in adipogenesis and is expressed prior to other proteins during early adipocyte differentiation. Among the three isoforms (PPAR α, β/δ, and γ) in the PPAR family, PPARγ is most specific to fat cells and exerts the strongest effect in adipogenesis. It is mainly expressed in adipose tissues and plays an important role in lipid metabolism and the adipocyte differentiation process. Previous studies have confirmed that the expression of PPARγ during differentiation induces the adipose phenotype, which is defined by lipid accumulation and the expression of other genes related to this process [8]. Additionally, PPARγ is known to be important in lipogenesis regulation, fatty acid metabolism, insulin-mediated glucose transport, and lipid oxidation [9]. Thus, a greater understanding of PPARγ expression and regulation is critical for understanding obesity and metabolic syndrome (MS). Much attention has been devoted to understanding pharmacologic activation of PPARγ [10]. However, the regulation of PPARγ expression, especially in the early stages of adipose commitment, is largely unknown. Studies on the transcription factors that control PPARγ expression in adipose progenitors may provide insight into adipocyte maturation in normal and obesity states.

Twist 1, which belongs to the basic helix-loop-helix family, is a well-conserved transcription factor that plays a role in the formation of a variety of tissues, including adipose tissue. Overexpression of Twist 1 has been confirmed in human brown adipose tissue (BAT) and white adipose tissue (WAT); however, the role of Twist 1 in obesity is not well defined. Pettersson et al. showed that Twist 1 might play a role in inflammation of human WAT by regulating the expression and secretion of inflammatory adipokines via direct transcriptional effects in white adipocytes [11]. Pan et al. found that Twist 1 interacted with PGC-1α, suppressed mitochondrial metabolism and uncoupling, and had an important role in the maintenance of energy homeostasis in human BAT [12]. The relationship between Twist 1 expression and the occurrence of obesity has gained attention in recent years. Twist 1 expression was shown to be downregulated in obese subjects and increased after weight loss based on abdominal subcutaneous WAT biopsy studies in 23 non-obese women and 107 obese women. The Twist 1 mRNA levels were correlated with adiponectin levels and inversely correlated with insulin resistance and adipocyte volume [13]. Further mechanisms of Twist 1 action in obesity development, especially with respect to the specific molecules involved in the underlying pathways, remain unexplored.

The present study was designed to (1) explore the relationship between Twist 1 expression and obesity in both diet-induced obese animal models and human subjects and (2) determine the role of Twist 1 in adipose differentiation and its potential relationship with the regulatory molecules that are involved in this process. We found that Twist 1 was negatively correlated with obesity development and that retroviral interference of Twist 1 expression did not impair the process of lipid formation in cultured 3T3-L1 preadipocytes; however, retroviral interference of Twist 1 altered the expression of PPARγ and influenced the secretion of multiple adipokines, mainly interleukins, growth factors, chemokines, and their receptors, thus providing a prospective mechanism linking Twist 1 expression with obesity or obesity-associated diseases.

## Results

### Verification of diet-induced obesity models in C57/BL6 mice and Wistar rats

**Figure 1:**
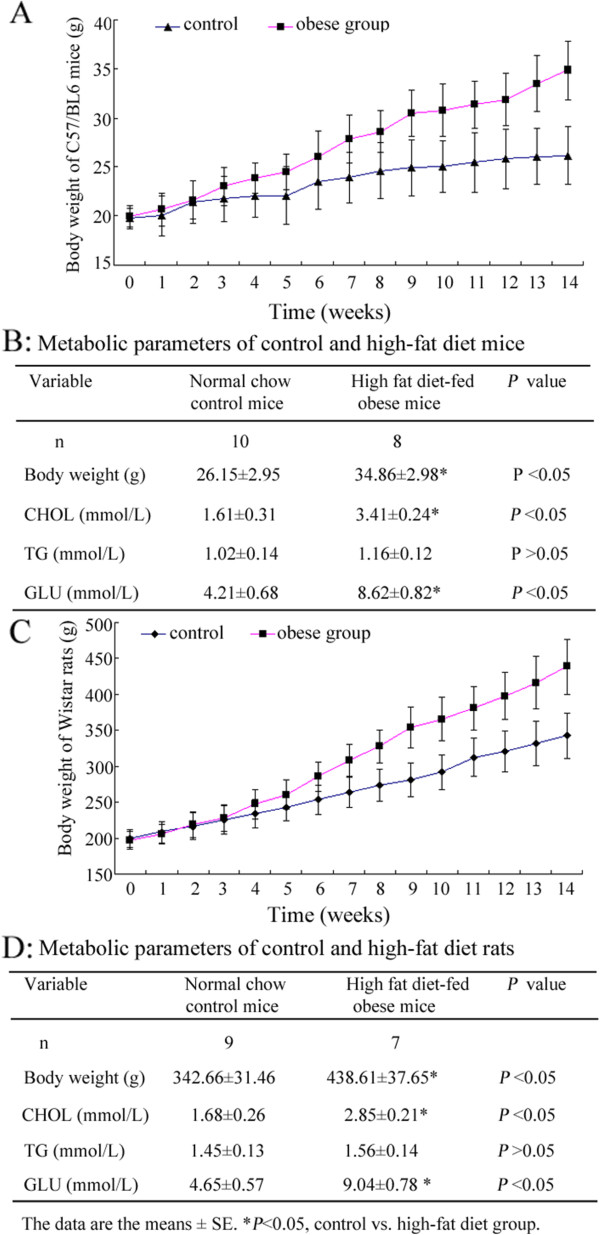
**Development and verification of diet-induced obesity models in C57/BL6 mice and Wistar rats. (A)** Change in the body weight of C57/BL6 mice during the development of the obesity model. **(B)** Serum CHOL, TG, and GLU levels of C57/BL6 mice were assayed. CHOL and GLU were obviously increased in obese mice compared to the control group (The data are presented as the mean ± SE. *P < 0.05, control vs. high-fat diet group). **(C)** Change in the body weight of Wistar rats during the development of the obesity model. **(D)** Serum CHOL, TG, and GLU levels of Wistar rats were assayed. CHOL and GLU were obviously increased in obese mice compared to the control (*P < 0.05, control vs. high-fat diet group).

During breeding, four C57/BL6 mice and two Wistar rats in the HFD-treated group died for unknown reasons, while all animals in the control group remained alive. Thus, there were 12 experimental animals in both of the control groups, eight C57/BL6 mice in the HFD-treated group, and ten Wistar rats in the HFD-treated group. During breeding, the body weights of C57/BL6 mice and Wistar rats increased gradually (Figure 1A/C). At the end of the experiments, the body weight of obese C57/BL6 mice was 34.86 ± 2.98 g, which was significantly higher than that of the control mice (26.15 ± 2.95 g; *P* < 0.05). The body weight of obese Wistar rats was 438.61 ± 37.65 g, while that of the control rats was 342.66 ± 31.46 g (*P* < 0.05). Serum CHOL and GLU levels were both obviously increased in obese mice and rats compared with the control (Figure 1B/D) (*P* < 0.05). However, no significant difference in TG levels was observed (*P* > 0.05).

### *Twist 1*gene transcription was significantly downregulated in obese subjects

**Figure 2:**
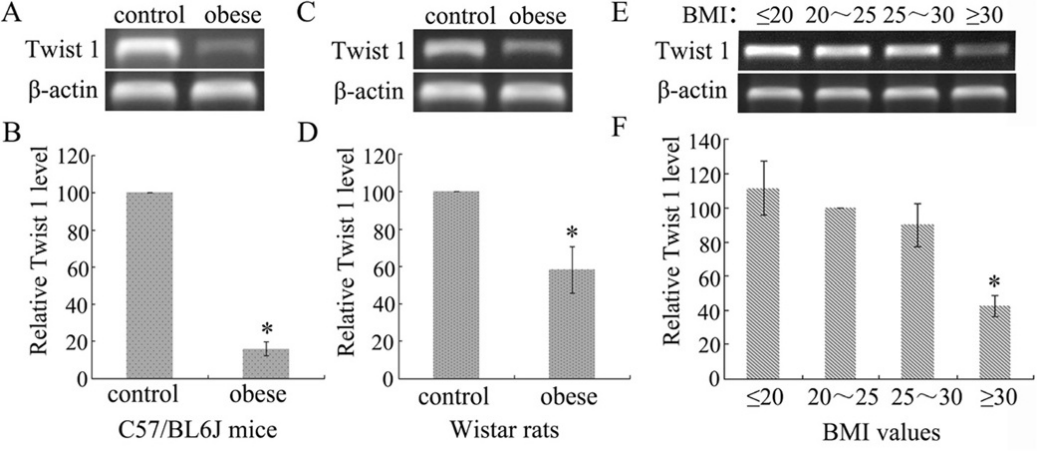
**Twist 1 transcription was downregulated in the adipose tissue of obese subjects. (A/B)** An obesity model was established in C57/BL6J mice that were fed a high-fat diet for 14 weeks. Twist 1 transcription was downregulated significantly in subcutaneous adipose tissue compared with its level in the control groups (*P < 0.05 vs. control). **(C/D)** An obesity model was established in Wistar rats that were fed a high-fat diet, and a reduced Twist 1 mRNA level was observed in subcutaneous adipose tissue (*P < 0.05 vs. control). **(E/F)** The level of Twist 1 transcription in the subcutaneous adipose tissues of individuals with a BMI ≥ 30 was lower than in non-obese individuals (*P < 0.05 vs. 20 < BMI < 25 group).

In both C57/BL6 mice (Figure 2A/B) and Wistar rats (Figure 2C/D), *Twist 1* gene transcription was significantly decreased in obese animals compared with non-obese animals. A semi-quantitative analysis using Image J software verified this decrease (*P* < 0.05). We further confirmed this decrease in *Twist 1* in clinically obese patients with a BMI ≥ 30 compared with non-obese patients (Figure 2E/F) (*P* < 0.05).

### Twist 1 expression was significantly downregulated in obese subjects

**Figure 3:**
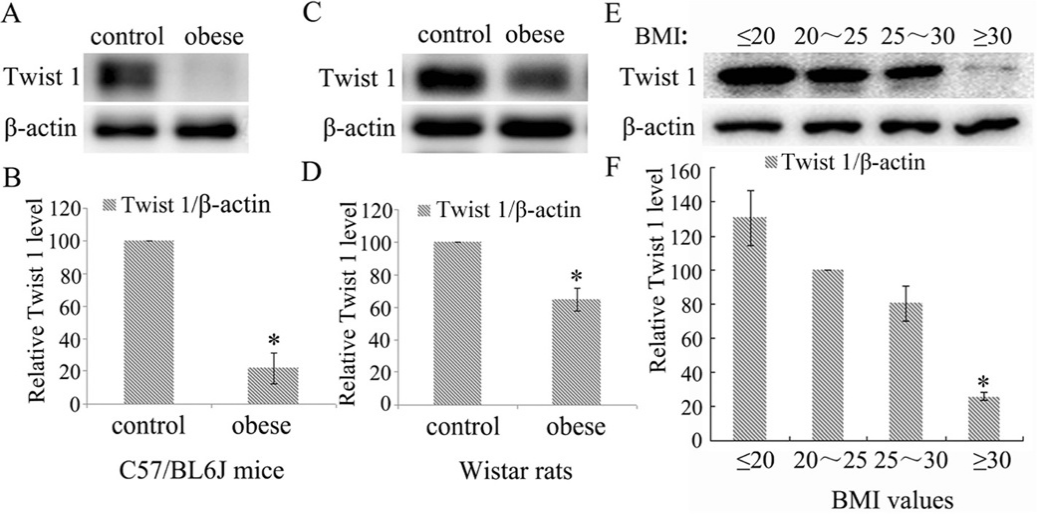
**Twist 1 protein expression was decreased in the adipose tissue of obese subjects. (A/B)** The expression of Twist 1 in subcutaneous adipose tissue from obese C57/BL6J mice was significantly downregulated compared with its expression in the control groups (*P < 0.05 vs. control). **(C/D)** Twist 1 expression was reduced in subcutaneous adipose tissues from obese Wistar rats (*P < 0.05 vs. control). **(E/F)** The expression level of Twist 1 in subcutaneous adipose tissues from individuals with a BMI ≥ 30 was shown to be lower than the level in non-obese individuals (* vs. 20 < BMI < 25 group, *P* < 0.05).

In C57/BL6 mice (Figure 3A/B), Wistar rats (Figure 3C/D), and clinical patients (Figure 3E/F), Twist 1 protein expression was significantly downregulated in obese subjects compared with relatively non-obese subjects based on western blot analysis (*P* < 0.05).

### Twist 1 was upregulated during 3T3-L1 adipocyte differentiation

**Figure 4:**
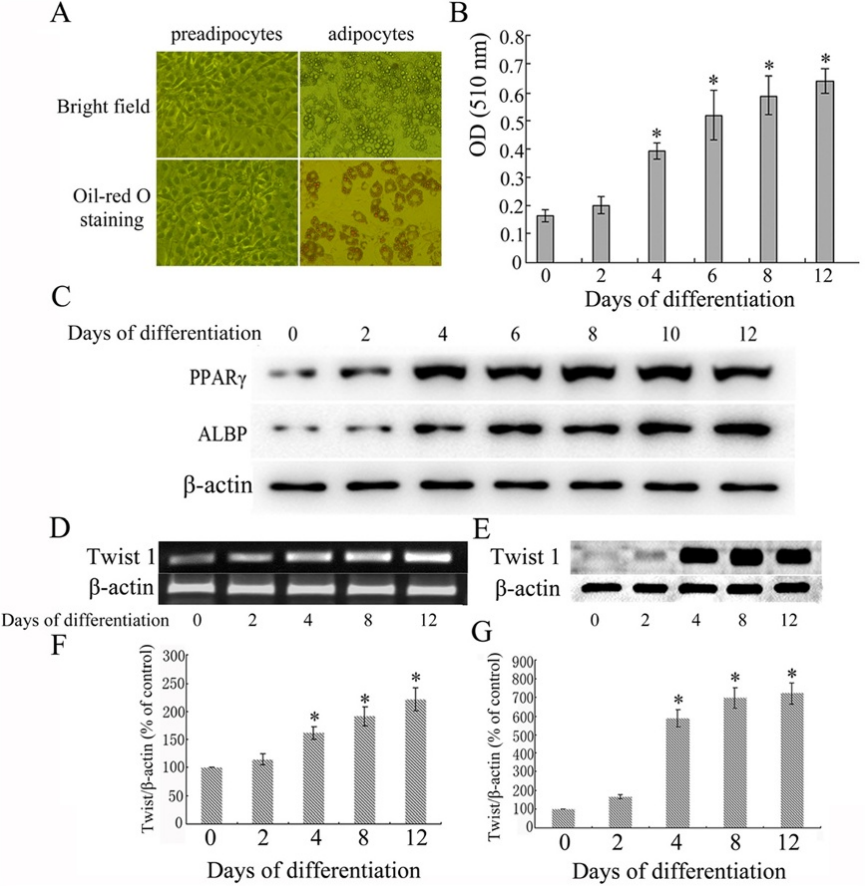
**3T3-L1 preadipocytes were induced to differentiate in vitro, and the dynamic expression of Twist 1 was detected. (A)** Morphological observations and oil red O staining of 3T3-L1 preadipocytes and adipocytes. **(B)** Quantitative analysis of intracellular oil red O staining using spectrophotometry. **(C)** PPARγ and ALBP protein expression levels at different stages of 3T3-L1 preadipocyte differentiation. **(D/F)** The dynamic transcription of Twist 1 was detected at different stages of 3T3-L1 preadipocyte differentiation (*P < 0.05 vs. control, day 0 cells). **(E/G)** The dynamic expression of Twist 1 was detected at different stages of 3T3-L1 preadipocyte differentiation (*P < 0.05 vs. control, day 0 cells).

Morphological observations revealed that the lipid droplets were distributed as a ring in the cytoplasm. Oil red O staining showed clear red dye in the adipocytes; however, this dye was not observed in the preadipocytes (Figure 4A). Quantitative analysis of the intracellular oil red O by spectrophotometry demonstrated the accumulation of lipids (Figure 4B). The levels of PPARγ and ALBP were significantly increased beginning at day 2 post-differentiation induction, and the levels peaked on day 12 (Figure 4C) (*P* < 0.05).

The dynamic changes in Twist 1 expression were measured by collecting cells at different time points during differentiation, including days 0, 2, 4, 8, and 12. The results demonstrated the upregulation of Twist 1 mRNA (Figure 4D/F) and protein (Figure 4E/G) beginning on the 4^th^ day of differentiation induction (*P* < 0.05).

### Retroviral interference of Twist 1 expression had no significant effect on lipid formation in 3T3-L1 preadipocytes

**Figure 5:**
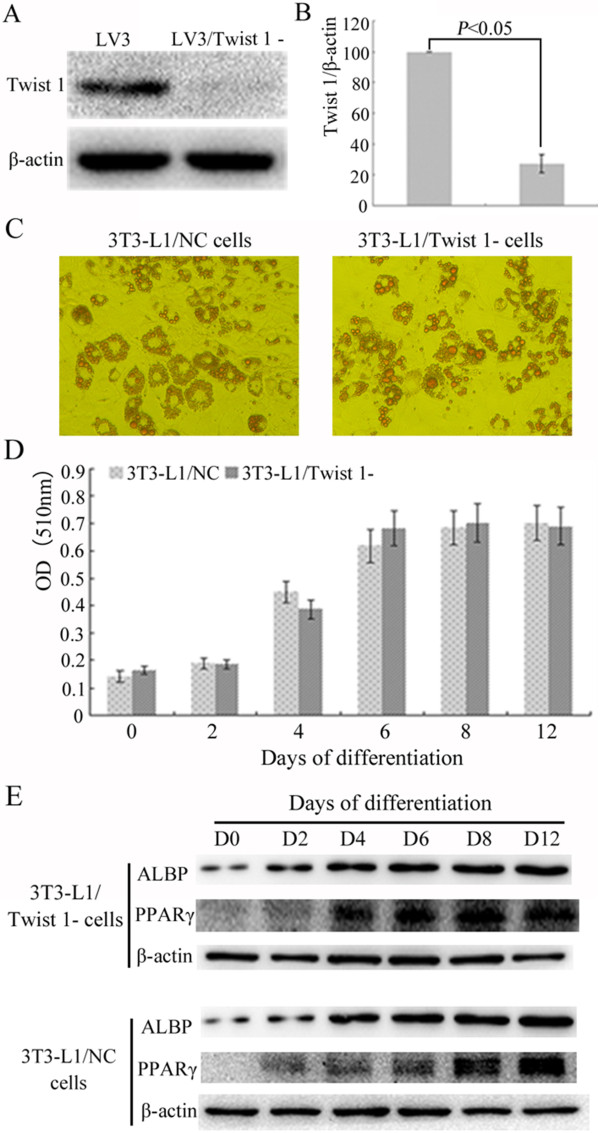
**The role of Twist 1 in adipogenesis was explored using retroviral interference of Twist 1 expression. (A/B)** Retroviral interference of Twist 1 expression was conducted using LV3. Western blot analysis showed that Twist 1 expression was reduced to 70-80% of the control. **(C/D)** Oil red O staining was used to analyze the differentiation of 3T3-L1/NC and 3T3-L1/Twist 1- cells. The results showed that interference of Twist 1 did not influence the differentiation, and there were no significant differences among the groups. **(E)** The expression levels of the PPARγ and ALBP proteins were increased in the Twist 1 shRNA-treated cells compared with the vector-treated control cells under hormone-induced differentiation. The infection time for both Twist 1/shRNA and LV3/NC was 48 h.

Western blot analysis demonstrated that the expression of Twist 1 was 70-80% reduced in Twist 1 shRNA-treated cells compared with its expression in vector-treated control cells (Figure 5A/B). When induced to differentiate, the Twist 1 shRNA-treated cells showed no obvious differences in intracellular lipid accumulation (*P* > 0.05) (Figure 5C/D). The expression levels of the PPARγ and ALBP proteins were increased in the Twist 1 shRNA-treated cells compared with the vector-treated control cells under hormone-induced differentiation. (Figure 5E).

### Retroviral interference of Twist 1 expression enhanced the expression of PPARγ on day 4 of hormone-induced differentiation

**Figure 6:**
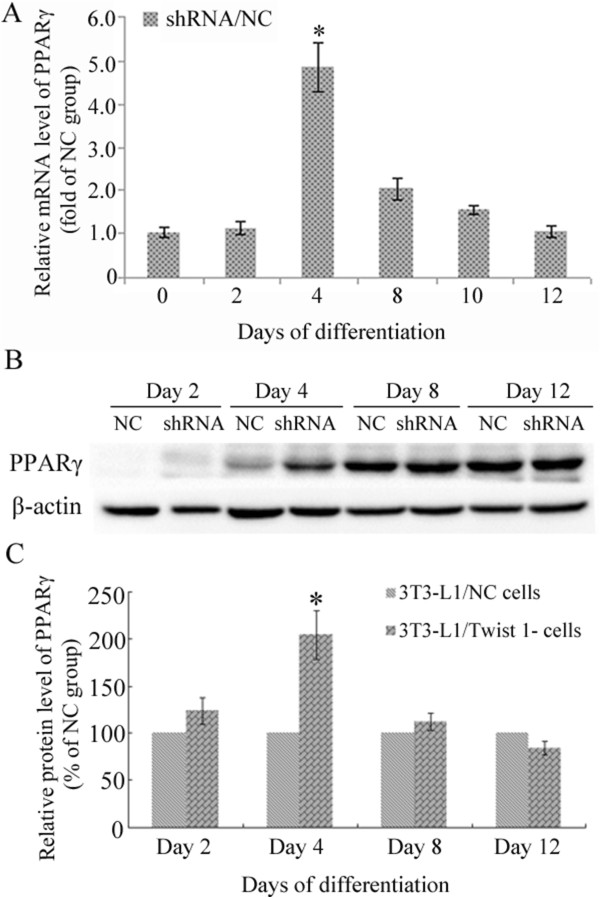
**Retroviral interference of Twist 1 expression enhanced the expression of PPARγ on day 4 of hormone-induced differentiation. (A)** Real-time PCR analysis revealed that PPARγ mRNA was upregulated on day 4 of hormone-induced differentiation after retroviral interference of Twist 1 expression compared with its expression level in 3T3-L1/NC cells (*P < 0.05 vs. day 0). **(B/C)** PPARγ protein expression on day 4 of hormone-induced differentiation was significantly enhanced when Twist 1 expression was downregulated; there were no obvious differences at the other time points (*P < 0.05 vs. 3T3-L1/NC cells at day 4). The infection time for both Twist 1/shRNA and LV3/NC was 48 h.

The mRNA level of PPARγ was assayed by real time PCR, and the results are expressed as the fold change compared to the control. The data were analyzed using the 2-^ΔΔ^ Ct method. The results confirmed the upregulation of PPARγ on day 4 of differentiation induction (*P* < 0.05); however, there were no obvious differences at the other time points (*P* > 0.05) (Figure 6A). A western blot assay confirmed the increased expression of PPARγ on day 4 of differentiation induction (*P* < 0.05), although there was no obvious difference at the other time points (*P* > 0.05) (Figure 6B/C). These results were consistent with the results of the real-time PCR.

### Adipokine array analyses showed that the secretion of multiple adipokines was altered by Twist 1 siRNA retroviral interference in 3T3-L1 preadipocytes

**Figure 7:**
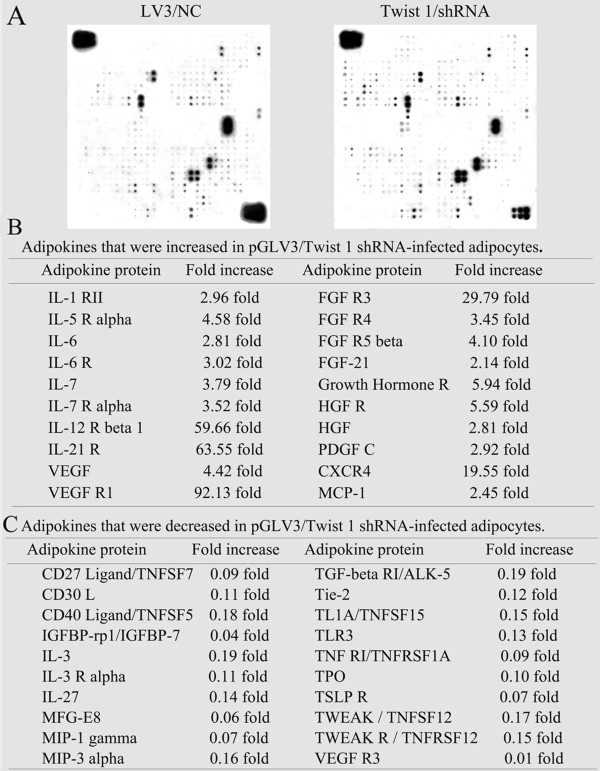
**The secretion of various adipokines by cultured 3T3-L1 adipocytes with or without Twist 1 interference was evaluated using a Mouse Antibody Array. (A)** Data from the array analyses with filter 1, which was treated with medium from the adipocytes infected with LV3/NC for 48 h (Left), and filter 2, which was treated with medium from the adipocytes infected with Twist 1/shRNA for 48 h (Right). **(B)** The values obtained from the scans were adjusted based on the intensity of the control spots on the filter corners, and the elevated levels of specific adipokines are shown. **(C)** Adipokines that were decreased by more than five fold in the medium of Twist 1-knockdown cells are listed.

As shown in Figure 7, the levels of twenty adipokines were increased by more than two-fold in the media of the Twist 1 shRNA-treated cells, including vascular endothelial growth factor receptor 1 (VEGFR1, 92.13-fold), interleukin 21 receptor (IL-21 R, 63.55-fold), interleukin 12 receptor beta 1 (IL-12 R beta 1, 59.66-fold), Cys-X-Cys receptor 4 (CXCR4, 19.55-fold), fibroblast growth factor receptor 3 (FGF R3, 29.79-fold), and growth hormone receptor (GHR, 5.94-fold) (Figure 7B).The levels of a total of fifty-seven adipokines were decreased. There was a greater than 5-fold change observed in the adipokines shown in Figure 7C, including VEGFR3 (0.01-fold), thymic stromal lymphopoietin receptor (TSLP R, 0.071-fold), macrophage inflammatory protein 1 gamma (MIP-1 gamma, 0.069-fold), TNF RI/TNFRSF1A (0.09-fold), milk fat globule EGF factor 8 (MFG-E8, 0.06-fold), IGFBP-rp1/IGFBP-7 (0.04-fold), and CD27 ligand/TNFSF7 (0.09-fold).

## Discussion

In this study, we provide evidence indicating that a molecular mechanism links Twist 1 expression to obesity or associated diseases. We found that retroviral interference of Twist 1 expression induced the expression of PPARγ during differentiation induction and increased the secretion of multiple adipokines into the medium; however, interference of Twist 1 expression did not significantly impair the process of lipid formation, indicating a potential role for Twist 1 in obesity and associated diseases.

Increasing evidence indicates that Twist 1 plays an important part in the development of obesity [14]. Previous studies have demonstrated that Twist 1 expression was decreased in subcutaneous WAT from 107 obese women compared with 23 non-obese (non-ob.) subjects, and the expression of Twist 1 was restored after weight loss [13]. The results presented here also revealed the downregulation of Twist 1 in obese individuals compared with non-obese individuals; these results are consistent with the results of several earlier studies. Regarding the species-organ expression of Twist 1, our analysis was conducted based on different species obesity models, including Wistar rats, C57/BL6 mice, and humans with different BMI values; these studies confirmed the decrease in Twist 1 levels in obesity.

Some recent studies have examined the molecular mechanism underlying the role of Twist 1 in obesity; however, the subject remains largely unexplored. In this study, we demonstrated that Twist 1 mRNA and protein levels were upregulated during 3T3-L1 differentiation; these results were similar to those described in previous reports on human preadipocyte differentiation in vitro [11]. No significant difference in differentiation was found between preadipocytes with Twist 1 interference and vector control cells; however, the role of Twist 1 in cell differentiation has been demonstrated in the dental pulp of extracted human third molars (DPSCs), and the forced expression of Twist 1 in DPSCs has been shown to alter the potential of these cells to differentiate into odontoblast-like cells [15]. Coincidentally, retroviral overexpression of Twist 1 in a brown fat preadipocyte cell line and in white fat preadipocyte 3T3-L1 cells had no significant effect on adipogenic differentiation in a previous study using oil red O staining analyses [12]. We examined lipid accumulation using oil red O staining; however, we also determined the expression levels of differentiation marker genes, including PPARγ and ALBP, due to the multiple confounding factors related to oil red O staining.

We determined the expression of PPARγ in preadipocyte clones, with or without Twist 1 interference, during differentiation induction. No prominent differences were observed in the different differentiation periods; however, comparisons at the same time points revealed an interesting result. Compared with the retroviral vector-transformed control cells, retroviral interference of Twist 1 expression in 3T3-L1 preadipocytes upregulated the mRNA and protein levels of PPARγ on day 4 of differentiation induction. The reason for this difference was unclear; however, this observation may indicate multiple possibilities due to the complex and diverse role of PPARγ and might provide a better understanding of the molecular basis underlying the important properties of Twist 1 as a target for obesity and associated diseases. For example, Twist 1 might influence the physiological and pathological processes related to PPARγ during the development of obesity. A previous study showed that PPARγ was important in brown fat metabolism, along with some other transcription factors including PPARα, ERRα, NRF1, and PGC-1α. Twist-1 is believed to suppress PGC-1α activity during this process by direct interaction through the C-terminal region of PGC-1α (aa 353–797). This protein-protein interaction of PGC-1α with Twist 1 may not be independent of PPARγ [16]. A regulatory cascade involving PPARγ and TWIST1 was found in low-grade chronic inflammation in humans, which is a major characteristic of obesity and results from deregulated white adipose tissue function. Treatment of diabetic obese patients with pioglitazone, an antidiabetic and anti-inflammatory PPARγ agonist, restored the expression of TWIST1 in adipose tissue [17]. Our results suggested a possible mechanism underlying the role of Twist 1 in obesity that is based on the multiple biological functions of PPARγ. PPARγ may therefore serve as an important molecular bridge between Twist 1 and obesity.

The levels of adipokines in the conditioned medium of 3T3-L1/Twist 1- cells were determined. Traditionally, adipokines are detected using enzyme-linked immunosorbent assays (ELISAs); however, the sensitivity and variability of this assay limit its application. In recent years, adipokine antibody arrays have become popular because this method can simultaneously detect multiple adipokines [18–20]. In the current study, a RayBio® Biotin Label-based Mouse Antibody Array was used to detect 308 adipokines related directly or indirectly to Twist 1 expression with high specificity. The results showed changes in the secretion of multiple adipokines, and the targeted adipokines were mainly divided into three categories. The first group consisted of interleukins (including IL-3, IL-6, IL-7, IL-9, IL-10, IL-17, IL-21, and IL-27) and their receptors. Studies exploring the roles of interleukins in obesity or associated diseases have been reported [21–24]. A lack of the interleukin-1 receptor I (IL-1 RI) was demonstrated to ameliorate high-fat diet (HFD)-induced insulin resistance (IR) by attenuating adipose tissue inflammation [25]. IL-1β is thought to support ectopic fat accumulation in hepatocytes and adipose-tissue macrophages by promoting adipose inflammation and limiting fat tissue expandability, contributing to impaired fat-liver crosstalk in nutritional obesity [26]. In obesity-associated asthma, the development of airway hyperreactivity (AHR) is thought to be dependent on IL-17A and the NLRP3 inflammasome [27]. Here, we extended the knowledge regarding the function of the IL family in Twist 1-related obesity and associated diseases.

The second group consisted of growth factors and their receptors, especially FGF, GHR, HGF, VEGF, TNF, and their receptors. The roles of several of these molecules in obesity have been described [28–32]; however, the importance of these molecules to Twist 1-related obesity remains unknown. The third group consisted of chemokine family members and related proteins, including CXCR4, Cys-Cys receptor 6 (CCR6), and monocyte chemotactic protein-1 (MCP-1). These results are consistent with the results of recent studies [13, 33–37]. There is clear evidence for the participation of the chemokine system in the development of obesity and obesity-induced pathologies [38]. However, much remains unknown about their roles in Twist 1-related obesity.

**Figure 8:**
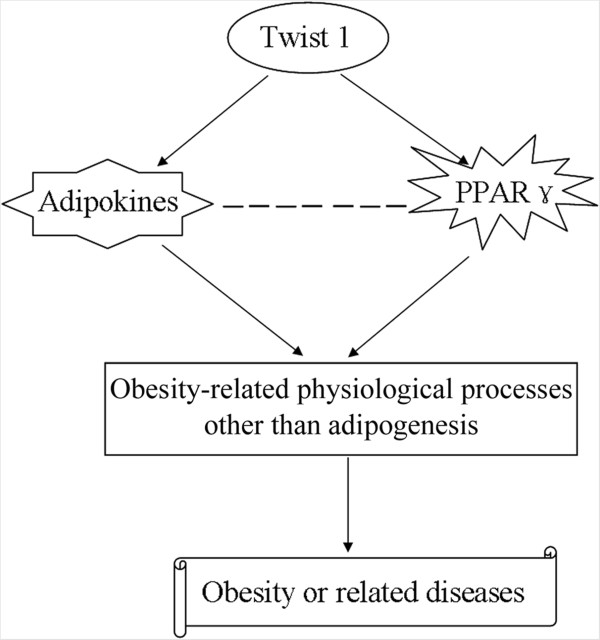
**A model for possible role of Twist 1 in obesity or obesity-related diseases.** The expression of Twist 1 influences the transcription and protein expression of PPARγ during differentiation induction and alters the secretion of multiple adipokines into the medium, either directly or indirectly. Then, altered PPARγ or adipokines play roles in obesity-related physiological process, but not in adipogenesis, which further regulates obesity or obesity-related diseases.

This study had some limitations. For example, it is not clear whether Twist 1 directly regulates PPARγ expression or how PPARγ acts as a molecular bridge among the Twist 1-related processes in obesity. The pathway from Twist 1 expression to alterations in the secretion of multiple adipokines is also not clear. Repeated analyses of the adipokine array are important; however, these were not completed in the current study because of funding limitations. These limitations indicate potential avenues for our future studies.We showed that retroviral interference of Twist 1 expression induced the expression of PPARγ during differentiation induction and altered the secretion of multiple adipokines, including interleukin, growth factors, chemokines, and their receptors; however, the interference did not significantly impair the process of lipid formation. These results indicate the existence of a potential mechanism linking Twist 1 expression and obesity or obesity-associated diseases (Figure 8).

## Materials and methods

### Materials

The animals, including C57/BL6 mice and Wistar rats, were purchased from the Experimental Animal Center of Shandong University. The 3T3-L1 mouse embryo fibroblasts were obtained from the American Type Culture Collection (ATCC, Washington, USA) (No. CL-173). Dulbecco’s modified Eagle’s medium (DMEM) was produced by the ATCC (No. 30–2002). Bovine serum and fetal bovine serum (FBS) were purchased from GIBCO (Invitrogen, California, USA). The RayBio® Biotin Label-based Mouse Antibody Array I was obtained from RayBiotech, Inc. (Norcross, GA). The primary antibodies anti-Twist 1, anti-PPARγ, anti-ALBP, and anti-β-actin were purchased from Abcam. All primers used in this study were synthesized at the Genomics Institute of HuaDa in Beijing. All other reagents, including insulin, dexamethasone (Dex), and isobutylmethylxanthine (IBMX), were purchased from Sigma (St. Louis, MO, USA).

### Obesity induction in C57/BL6 mouse and Wistar rat models and subsequent analyses

Twenty-four adult male C57/BL6 mice aged 6 weeks and twenty-four adult male Wistar rats aged 3 months were purchased from the Experimental Animal Center of Shandong University. The C57/BL6 mice were housed 6 to a cage, and the Wistar rats were housed 3 to a cage. The animals were maintained in a controlled environment at 25°C with 55% relative humidity under a 12/12-h light/dark cycle; they were provided free access to tap water and food. After one week of adaptation, the mice and rats were randomly divided into two groups with 12 animals per group: the “control group” (normal chow, 4% fat) and the “high-fat group” (high-fat diet, 20% fat). The basal and high-fat diet were both purchased from Beijing Ke Ao Xie Li Feed Co., Ltd, and detailed information regarding these diets has been published previously [39]. The body weight of each animal was determined once per week for 14 weeks until the animals were sacrificed by deep anesthesia with 0.3% pentobarbital sodium (1 ml/kg, intraperitoneal injection). The animal experiments were performed in accordance with the ‘Principles of Laboratory Animal Care’ established by the National Institutes of Health and were approved by the Animal Care and Use Committee of Shandong University.

Blood was collected in EDTA-coated tubes after fasting the mice for 12 h, and the serum was separated by centrifugation (500 × g, 4°C, 10 min). Serum cholesterol (CHOL), triglycerides (TG), and glucose (GLU) levels were measured using the Beckman DXC 800 analyzer (Beckman Coulter, Inc., California, USA). All biochemical measurements were carried out at the Department of Laboratory Medicine of Shandong Provincial Qianfoshan Hospital. Subcutaneous adipose tissue samples were collected and quickly frozen by immediate immersion into liquid nitrogen. They were then stored at -80°C prior to mRNA and protein extraction to determine Twist 1 mRNA and protein expression levels.

### Human fat sample collection

A total of 90 patients who underwent liposuction or fat transplant surgeries at Shandong Provincial Qianfoshan Hospital affiliated with Shandong University from 2011 to 2013 were recruited for this study. All patients were euthyroid, had no history of diabetes, and had no family history of obesity. Subcutaneous adipose tissue samples were collected. Written informed consent was obtained from all of the patients before surgery, and the study was approved by the Ethics Committee of Shandong University. The samples were divided into 4 groups based on the patient body mass index (BMI) (calculated as body weight (BW) in kilograms over squared height in meters): the slim group (15 cases, BMI ≤ 20); the normal group (26 cases, 20 < BMI < 25); the overweight group (21 cases, 25 ≤ BMI < 30); and the obesity group (28 cases, BMI ≥ 30). The basic clinical characteristics of these patients are shown in Table 1. The clinically relevant BMI divisions for Asians, in which obesity is a BMI greater than 27, were not applied here. Instead, we considered a BMI greater than 30 to be indicative of obesity. The collected adipose tissue samples were also analyzed to determine Twist 1 mRNA and protein expression.

**Table 1:** **Information regarding human samples collected from the clinic**

***BMI***	***Number***	***Sex***		***Types of surgery***	
		Male	Female	Liposuction	Fat transplant surgery
BMI ≤ 20	15	6	9	3	12
20 < BMI ≤ 25	26	8	18	9	17
25 < BMI ≤ 30	21	9	12	14	7
BMI > 30	28	11	17	22	6
Total	90	34	56	48	42

### Adipose tissue pretreatment

Lipids in adipose tissues interfere with PCR and western blot analyses. Therefore, removal of the majority of the triglycerides from the adipose tissues before further analyses was essential. We used pre-cooled acetone to dissolve the fat as previously described [39]. Importantly, we ensured that a sufficient amount of acetone was used for full lipid extraction, performed appropriate intermittent shaking during the incubation at 4°C, and minimized protein degradation by using protease inhibitors. After centrifugation, the lipid droplets appeared in the upper layer, while the other components of the cells remained in the bottom of the tube. The upper layers were removed and discarded as carefully as possible. The procedure was repeated if visible lipids remained.

### Preadipocyte differentiation induction and oil red O staining

The 3T3-L1 preadipocytes were maintained in DMEM supplemented with 10% bovine serum, 100 U/ml penicillin, and 100 mg streptomycin at 37°C in a humidified atmosphere composed of 95% air and 5% CO_2_. Preadipocyte differentiation induction and oil red O staining were conducted as previously described [39].

### Knockdown of Twist 1 in 3T3-L1 preadipocytes

The lentivirus vector pGLV-H1-GFP/Puro (pLV3) was used to generate the shRNA that targeted Twist 1 according to the manufacturer’s instructions. The oligo sequence of the siRNA targeting Twist 1 was 5’-ACTCCAAGATGGCAAGCTG-3’. Packaged viruses encoding an shRNA targeting Twist 1 based on the obtained plasmids (pLV3/Twist 1-shRNA) were applied to cultured 293T cells according to the manufacturer’s instructions. The blank pLV3 vector was used to generate virus without siRNA as a control. After infection for 48–72 h, the 293T cells were centrifuged, and the supernatants (containing virus) were used to infect 3T3-L1 cells once the cells had reached 70-80% confluence. Twenty-four hours post-infection, stable transfectants were selected based on puromycin resistance and green fluorescent protein (GFP) expression under fluorescence microscopy. The cells stably expressing the pLV3 vector were used as a control. The preadipocytes with Twist 1 expression interference and those expressing the pLV3 vector were induced to differentiate as described above, and adipocytes or conditioned media were collected for transcription, protein expression, and adipokine array analyses.

### RT-PCR and real-time PCR

Total RNA was extracted using TRIzol (Invitrogen) according to the manufacturer’s instructions. Complementary DNA (cDNA) was synthesized using the ExScript RT reagent kit (Takara Bio, Otsu, Shiga, Japan) following the manufacturer’s instructions. The detailed RT-PCR protocol used to analyze Twist 1 gene expression has been previously described, and β-actin was used as a control [39]. Real-time PCR was performed to quantify PPARγ mRNA using a CFX96TM Real-Time System (Bio-Rad) by monitoring the increase in fluorescence of SYBR Green, which is a double-stranded dye that specifically binds DNA. The real-time PCR reactions were carried out in a final volume of 20 μl, including 1 μl of cDNA sample, 1 μl of each primer, and 10 μl of 2x Ultra SYBR Mixture (product no. cw0956). The cycling conditions were 95°C for 10 min followed by 40 cycles of 95°C for 15 s, 60°C for 1 min, and 72°C for 30 s. The relative mRNA expression level of the target sequence was determined as the fold change compared with the control (β-actin mRNA) using the 2-ΔΔ Ct method or Image J software analysis. All measurements were conducted in triplicate. The PCR primers were designed using Primer 5.0 Software, and the sequences are as follows:

- Twist 1-F: 5’-CAACAGCGAGGAGGA-3’
- Twist 1-R: 5’-CGCCAGTTTGAGGGT-3’
- PPARγ-F: 5’-TGGTGACTTTATGGAGCCTAA-3’
- PPARγ-R: 5’-GGCGAACAGCTGAGAGGACTCTG-3’
- β-actin-F: 5’-GTGACGTTGACATCCGTAAAGA-3’
- β-actin-R: 5’-GCCGGACTCATCGTACTCC-3’

### Western blot analysis

Total protein was extracted using radio immunoprecipitation assay (RIPA) lysis buffer containing protease and phosphatase inhibitors according to the manufacturer’s instructions. The protein concentration was determined based on a Bradford protein assay. The proteins (80 μg from each sample) were resolved on SDS-PAGE gels, transferred to a Hybond-P PVDF membrane, and incubated with primary antibodies (anti-Twist 1 (1:1000), anti-PPARγ (1:1000), anti-ALBP (1:500), or anti-β-actin (1:2500)) overnight at 4°C. Twist 1, PPARγ, ALBP, and β-actin bands were visualized at apparent molecular weights of 21 kDa, 58 kDa, 15 kDa, and 43 kDa, respectively, by incubation with peroxidase-conjugated anti-rabbit or anti-mouse secondary antibodies (1:5000 dilution) for 1 h at room temperature. The relative OD ratio was calculated with Image J software by comparison with β-actin; data from three experiments were analyzed.

### Adipokine array

The ability of 3T3-L1 adipocytes to secrete various adipokines into conditioned media after Twist 1 interference was evaluated and compared with that of control cells using a RayBio® Biotin Label-based Mouse Antibody Array I obtained from RayBiotech, Inc. (Norcross, GA). The assay was performed according to the manufacturer’s protocol and could be used to simultaneously detect 308 adipokines with high specificity. Briefly, 2 ml of 1× blocking buffer was added to each membrane, and the membranes were incubated at room temperature for 30 min. The membranes were then incubated with 1 ml of the sample at room temperature for 2 h. The samples were decanted from each container and washed 3 times with wash buffer at room temperature with shaking for 5 min. Blocking buffer (1×, 100 μl) was added to the biotin-conjugated anti-adipokine tube. Diluted biotin-conjugated antibodies (1 ml) were added to each membrane. The membranes were then incubated at room temperature for 2 h and washed with washing buffer. Next, 2 ml of 1000-fold diluted HRP-conjugated streptavidin was added to each membrane. The membranes were incubated at room temperature for 2 h, washed with washing buffer, and exposed to X-ray film. The signal was detected using a film developer or directly on the membrane using a chemiluminescence imaging system.

### Statistical analysis

The data are presented as the mean ± standard error of the mean (SEM). Comparisons between the means of two groups were analyzed using a *t test*. One-way analysis of variance (ANOVA) was performed when there were more than 2 groups using the SPSS 16.0 software package. A *q test* was used for further pairwise comparisons. *P* values of less than 0.05 were considered statistically significant.
